# Transcriptomic resources for *Bagrada hilaris* (Burmeister), a widespread invasive pest of Brassicales

**DOI:** 10.1371/journal.pone.0310186

**Published:** 2024-12-27

**Authors:** Michael E. Sparks, David R. Nelson, Robert L. Harrison, Nicholas R. Larson, Daniel Kuhar, Ariela I. Haber, Sam D. Heraghty, Zarley Rebholz, Dorothea Tholl, Ian M. Grettenberger, Donald C. Weber, Dawn E. Gundersen-Rindal

**Affiliations:** 1 Invasive Insect Biocontrol and Behavior Laboratory, USDA-ARS, Beltsville, Maryland, United States of America; 2 Department of Microbiology, Immunology and Biochemistry, University of Tennessee Health Science Center, Memphis, Tennessee, United States of America; 3 Department of Biological Sciences, Virginia Tech, Blacksburg, Virginia, United States of America; 4 Department of Entomology and Nematology, University of California, Davis, Davis, California, United States of America; Zhejiang University, CHINA

## Abstract

The bagrada bug, *Bagrada hilaris* (Burmeister), is an emerging agricultural pest in the Americas, threatening agricultural production in the southwestern United States, Mexico and Chile, as well as in the Old World (including Africa, South Asia and, more recently, Mediterranean areas of Europe). Substantive transcriptomic sequence resources for this damaging species would be beneficial towards understanding its capacity for developing insecticide resistance, identifying viruses that may be present throughout its population and identifying genes differentially expressed across life stages that could be exploited for biomolecular pesticide formulations. This study establishes *B*. *hilaris* transcriptomic resources for eggs, 2^nd^ and 4^th^ larval instars, as well as male and female adults. Three gene families involved in xenobiotic detoxification—glutathione S-transferases, carboxylesterases and cytochrome P450 monooxygenases—were phylogenetically characterized. These data were also qualitatively compared with previously published results for two closely related pentatomid species—the brown marmorated stink bug, *Halyomorpha halys* (Stål), and the harlequin bug, *Murgantia histrionica* (Hahn)—to elucidate shared enzymatic components of terpene-based sex pheromone biosynthetic pathways. Lastly, the sequence data were screened for potential RNAi- and virus-related content and for genes implicated in insect growth and development.

## Introduction

The bagrada bug, *Bagrada hilaris* (Burmeister) (Hemiptera: Pentatomidae), also known as painted bug, is a pentatomid stink bug that is a major pest of cruciferous crops (Brassicaceae). This species exhibits a strikingly similar coloring pattern to that of the harlequin bug, *Murgantia histrionica* (Hahn), and shares this species’ specialization on crucifers. It is native to East and South Africa as well as South Asia [1–3]. In 2008, bagrada bug was reported in the USA in California and has since spread to the adjacent states of Arizona, New Mexico, Nevada, Utah and western Texas [4], as well as the Mexican states of Baja California, Chihuahua, Coahuila, Durango, Sonora and Sinaloa [5, 6]. It was also introduced into Hawaii [7], Chile [8], and the Mediterranean islands of Pantelleria, Italy and Malta [9]. Climatic projections show that the species could continue to invade large acreages of valuable croplands, particularly in areas with a Mediterranean climate such as Spain, Portugal, North Africa, Australia, Argentina and additional areas in Chile [10]. In the past four-to-eight years, for unknown reasons, populations in the USA and Mexico have decreased, reducing or eliminating the pest status of bagrada bug in these areas (I.M.G., unpublished; John C. Palumbo, University of Arizona, Tucson, AZ, USA, pers. comm., 20 Feb. 2023; Sergio Sanchez-Peña, Universidad Autónoma Agraria Antonio Narro, Saltillo, México, pers. comm., 22 Feb. 2023).

Although their hosts are primarily cruciferous plants such as cabbage, cauliflower and broccoli, bagrada bugs have also been historically observed to infest and damage a wide variety of crops, including cotton, potato, green beans, wheat and sorghum [11–14]. Due to their sporadically large populations and rapid colonization of susceptible crops, bagrada bug can kill or severely damage direct-seeded crops and seedlings, at times resulting in total yield loss. Growers in North America have typically relied on broad-spectrum insecticides to suppress local populations because alternative tactics have not been available [4]. Classical biological control based on foreign exploration in the bagrada bug’s native range [15] is currently being pursued, as is the potential use of sterile insect technique [16]. At the same time, a species of egg parasitoid from Pakistan that attacks bagrada bug has also spontaneously appeared in California [17].

Although exhibiting similar coloration patterns to the harlequin bug, bagrada bug adults are only about one fourth to one third the size of *M*. *histrionica* adults. Female adult bagrada bugs are slightly larger than male adults of the species. Their habit of laying single eggs in the soil is unusual for pentatomids and likely reduces attack by many natural enemies that search plants to identify their prey. Highly heat- and drought-tolerant, the bug completes its life cycle in 18 days at 30°C [14, 18], peak activity in adults is observed in a temperature range of 25°C to 41°C [14], and fecundity reaches 150 eggs per female [13]. It proceeds through five nymphal instar stages, although first instars are non-feeding [13].

The tribe Strachiini of the subfamily Pentatominae [19] contains several genera of stink bugs specializing on plants in the mustard family (Brassicaceae) and related plants: *Bagrada*, the New World *Murgantia* (including the harlequin bug, *M*. *histrionica*), and the Old World genera *Eurydema* [20] and *Stenozygum* [21, 22]. These bugs sequester and/or detoxify glucosinolates present in their plant hosts [23]. Bagrada bug feeding causes starburst-shaped chlorotic lesions through its lacerate-and-flush feeding method with piercing-sucking mouthparts [4, 24, 25]. Due to this cellular disruption of plant tissue, one would expect these insects to be biochemically competent at detoxification of isothiocyanates and other breakdown products from the glucosinolate-myrosinase plant defense system [26].

Understanding the mechanisms of xenobiotic detoxification is of particular interest in invasive and pest insects given that detoxification is implicated in susceptibility to insecticides [27], which can complicate management of the species. There are reports of insecticide resistance developing in bagrada bug in Italy [9], as well as in other stink bug species [28]. Generating transcriptomic resources is an important first step in beginning to understand how insecticide resistance might evolve in a particular species. What is more, such datasets would also be useful towards identifying viruses that may be present throughout bagrada bug populations, characterizing its sex pheromone biosynthetic pathways and identifying genes differentially expressed across life stages that could be exploited as knockdown targets in biomolecular pesticide formulations.

Concerning the phenomena of xenobiotic detoxification, three gene families have significant relevance to the evolution of insecticide resistance—glutathione S-transferases (GSTs), carboxylesterases (COEs) and cytochrome P450 monooxygenases (CYPs). These families have been evaluated in several other insect taxa in previous studies lead by the authors [29–31]. Each of these gene families is evolutionarily diverse and has been implicated in insecticide resistance for hemipteran pests [32]. They are primarily involved in Phases I and II of xenobiotic detoxification, in which xenobiotics are processed so they can be expelled in Phase III [33]. The Delta and Epsilon classes of GSTs have led to insecticide resistance and, unlike other GST classes, are only found in insects [34, 35]. The β-esterase class of the carboxylesterases has also been observed to confer insecticide resistance and exhibits considerable variation in copy number across Hemiptera [33]. Lastly, the cytochrome P450 family has been noted for its diverse functionality, with the CYP3 clan in particular exhibiting insecticide resistance and a quite dynamic evolutionary history [32, 36].

This study establishes *B*. *hilaris* transcriptomic resources for eggs, 2nd and 4th larval instars, as well as for male and female adults. The aforementioned gene families related to xenobiotic detoxification—glutathione S-transferases, carboxylesterases and cytochrome P450 monooxygenases—were phylogenetically characterized. The sequence data were qualitatively compared with previously published results for two closely related pentatomids—the brown marmorated stink bug, *Halyomorpha halys* (Stål), and the harlequin bug, *Murgantia histrionica* (Hahn) [30]—to elucidate shared enzymatic components of terpene-based sex pheromone biosynthetic pathways. The data were also screened for potential RNAi- and virus-related content and for genes implicated in insect growth and development.

## Materials and methods

### Insect samples

Bagrada bugs were obtained from the University of California, Davis laboratory rearing, originally collected in King City and San Ardo, Monterey County, California. These were maintained in a growth chamber in Beltsville under APHIS PPQ permit P526P-17-02011 and held at 27°C with relative humidity ~20% under continuous combined fluorescent and incandescent light. The insects were fed certified organic broccoli florets 3x weekly and not provided any other water or food source. They were housed in clear polystyrene containers (27 x 20 x 8.5 cm or 19.5 x 14 8.5 cm; Tri-State Plastics, Latonia, Kentucky), ventilated using stainless-steel screen (mesh 2.5 per mm, with square openings of 0.3 mm, TWP Inc., Berkeley, California). Open 9 cm diameter polystyrene Petri dishes (Falcon, ThermoFisher Scientific, Waltham, Massachusetts) filled with fine sand (Décor Sand, Activa Products, Marshall, Texas) to an approximate depth of 5 mm provided an oviposition substrate. These were placed under cut-away paper cups (Solo 235 ml, paper with double-sided polyethylene coating, KHB8A J8000, Solo Cup Co., Lake Forest, Illinois) to simulate shade adjacent to a plant. Eggs were harvested by sifting the sand through a #30 (0.6 mm) screen three times weekly. They were then placed into a smaller rearing container (300 ml transparent styrene-acrylonitrile box with opaque polyethylene top, Mepal BV, Lochem, Netherlands, provided with two 23 mm side holes covered with screening) and provisioned only cut broccoli stems until the third instar, transitioning to broccoli florets for later nymphs and adults.

### Transcriptome sequencing, assembly, expression analysis and annotation

Three biological replicates apiece were sequenced for each of eggs, 2^nd^ and 4^th^ nymphal instars, as well as approximately seven-day-old unmated male and female adults, using Illumina PE150. Replicates for nymphs and adults consisted of 15 pooled individuals apiece; 20 eggs were pooled for each egg replicate. Sequencing volumes achieved are presented in Table 1. Reads were pooled, digitally normalized and globally assembled using version 2.6.6 of the Trinity program [37]. Raw sequencing reads and assembled transcripts are publicly available at the NCBI SRA and TSA divisions, respectively, under BioProject PRJNA854805. Gene expression levels were estimated using version 0.11.3 of salmon [38], the results of which were processed with DESeq2 (v1.36.0) [39] to identify differentially expressed genes (DEGs) in each of the ten possible pairwise comparisons among the five categorical levels described above. Specifically, a gene was identified as differentially expressed in a comparison if it exhibited a false discovery rate of not more than 0.05 and at least a doubling of mean abundance between categorical levels. To complement gene-level, alignment-free expression analysis results obtained from salmon and DESeq2, reads were also aligned to assembled mRNA pseudomolecules using bowtie 2 (v2.3.4.1) [40] and processed with RSEM (v1.3.3) [41], thereby producing both gene- and transcript-level expression estimates (conveyed using the transcripts per million measure, TPM [42]). Transcripts were compared with the NCBI NR protein database using the BLASTx-like alignment tool, DIAMOND (v2.0.4), with default parameter settings [43]. Protein family annotations for transcripts were prepared by comparison with the Pfam protein database [44] using HMMER (v3.3.4) with default parameter settings [45]; GO terms were extracted from Pfam hits by means of the pfam2go table provided by the Gene Ontology knowledgebase [46, 47].

**Table 1:** Raw sequencing volumes achieved for each developmental stage and/or sex considered, for each biological replicate. A length of 150bp was targeted for each paired read. Reads were quality trimmed in advance by the sequencing vendor.

	Egg Mass		
	*biorep 1*	*biorep 2*	*biorep 3*
read pairs	152,928,356	121,536,304	136,001,824
bases	46,184,363,512	36,703,963,808	41,072,550,848
	2^nd^ Instar Nymphs		
	*biorep 1*	*biorep 2*	*biorep 3*
read pairs	31,487,688	43,463,866	39,062,495
bases	9,509,281,776	13,126,087,532	11,796,873,490
	4^th^ Instar Nymphs		
	*biorep 1*	*biorep 2*	*biorep 3*
read pairs	38,432,688	36,908,027	41,142,831
bases	11,606,671,776	11,146,224,154	12,425,134,962
	Male Adults		
	*biorep 1*	*biorep 2*	*biorep 3*
read pairs	37,742,349	50,255,031	50,168,228
bases	11,398,189,398	15,177,019,362	15,150,804,856
	Female Adults		
	*biorep 1*	*biorep 2*	*biorep 3*
read pairs	42,621,118	47,393,334	44,498,428
bases	12,871,577,636	14,312,786,868	13,438,525,256

### Glutathione S-transferase gene family analysis

**Fig 1:**
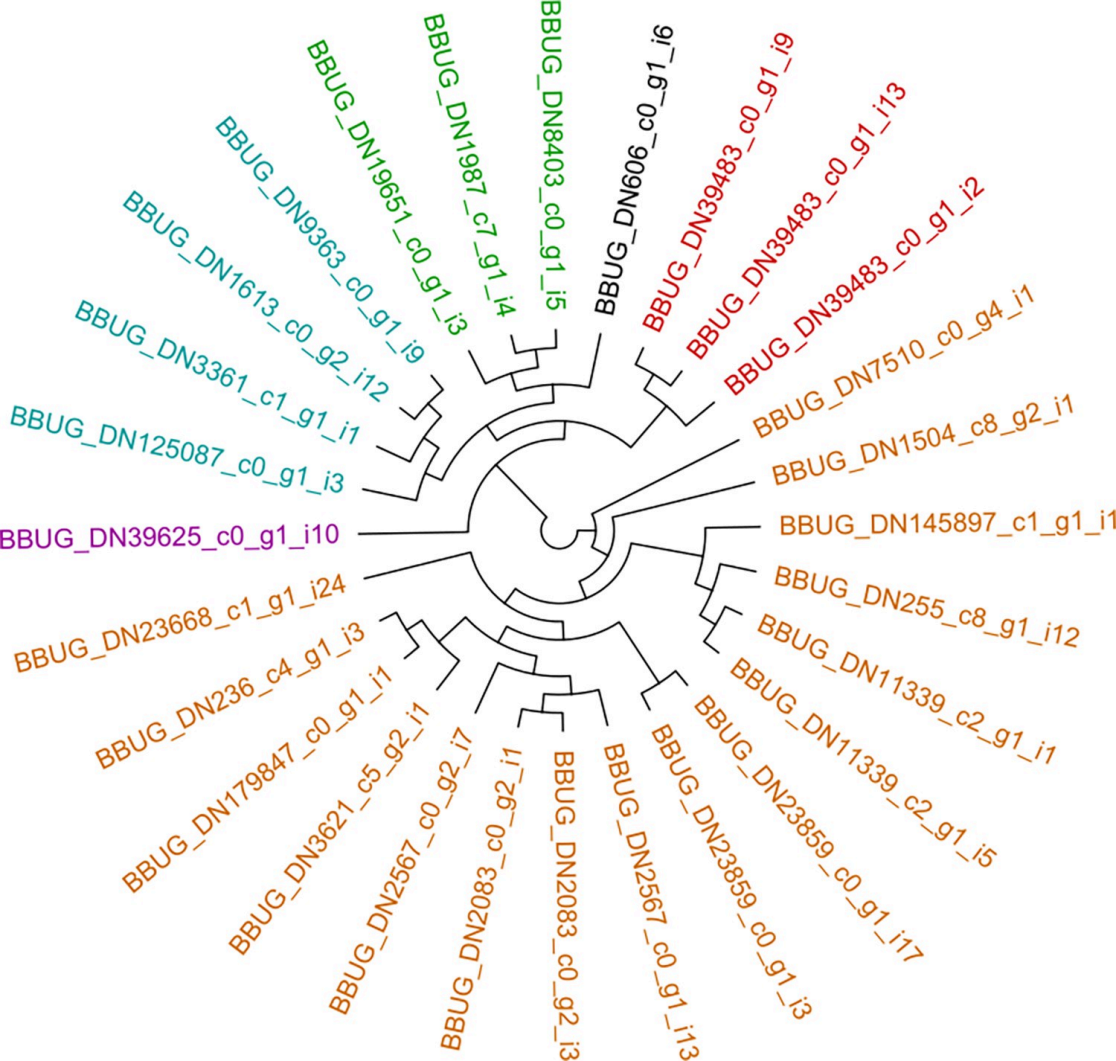
Glutathione S-transferase (GST) enzymes from *B*. *hilaris*. Cladogram of bagrada bug GSTs distilled from the comprehensive, ten-taxa phylogram presented in S1 Fig. GST classes are connoted by leaf coloring: turquoise ~ Theta, green ~ microsomal, brown ~ Sigma, purple ~ prostaglandin E synthase, red ~ Delta, and black ~ not classified. (No Omega- or Epsilon-class sequences were observed in bagrada bug).

A set of 102 reference glutathione S-transferase (GST) protein sequences, identified using prior annotation information, was obtained from four hemipteran species: *Halyomorpha halys* (brown marmorated stink bug) = 35 sequences, *Murgantia histrionica* (harlequin bug) = 32, *Cimex lectularius* (bed bug) = 15 and *Diaphorina citri* (Asian citrus psyllid) = 20. The *B*. *hilaris* transcriptome was compared against these using tBLASTn [48] with default parameters, which identified 28 bagrada bug GST instances. To enable class-specific comparisons between hemimetabolous and holometabolous insects, these hemipteran GSTs were combined with 189 coleopteran GST sequences identified in Sparks et al. (2020) [29]: *Anoplophora glabripennis* (Asian long-horned beetle) = 37 sequences, *Leptinotarsa decemlineata* (Colorado potato beetle) = 30, *Acalymma vittatum* (striped cucumber beetle) = 43, *Tribolium castaneum* (red flour beetle) = 37 and *Diabrotica virgifera virgifera* (western corn rootworm) = 42. The total set of 319 insect GSTs (available in Supplementary Data as “SuppFile1.GST.fpa.txt”; Supplementary Data are provided in the zipped archive file, “BBUG_SuppInfo_11Dec2024.zip”, made publicly available from the Open Science Framework repository at https://doi.org/10.17605/OSF.IO/8W3HA) was multiply aligned at the protein level using MUSCLE v3.8.31 [49], the results of which were used to calculate a maximum likelihood tree with RAxML v1.1.0 [50]. The resulting phylogeny was rendered using FigTree [51] (see S1 Fig). Coleopteran genes were purged from the comprehensive phylogeny using newick-tools v0.0.1 [52] to produce a Hemiptera-only GST phylogram (see S2 Fig). Similarly, *B*. *hilaris* nodes were distilled from the composite phylogeny, the branching pattern of which is presented in the cladogram of Fig 1.

### Carboxylesterase gene family analysis

**Fig 2:**
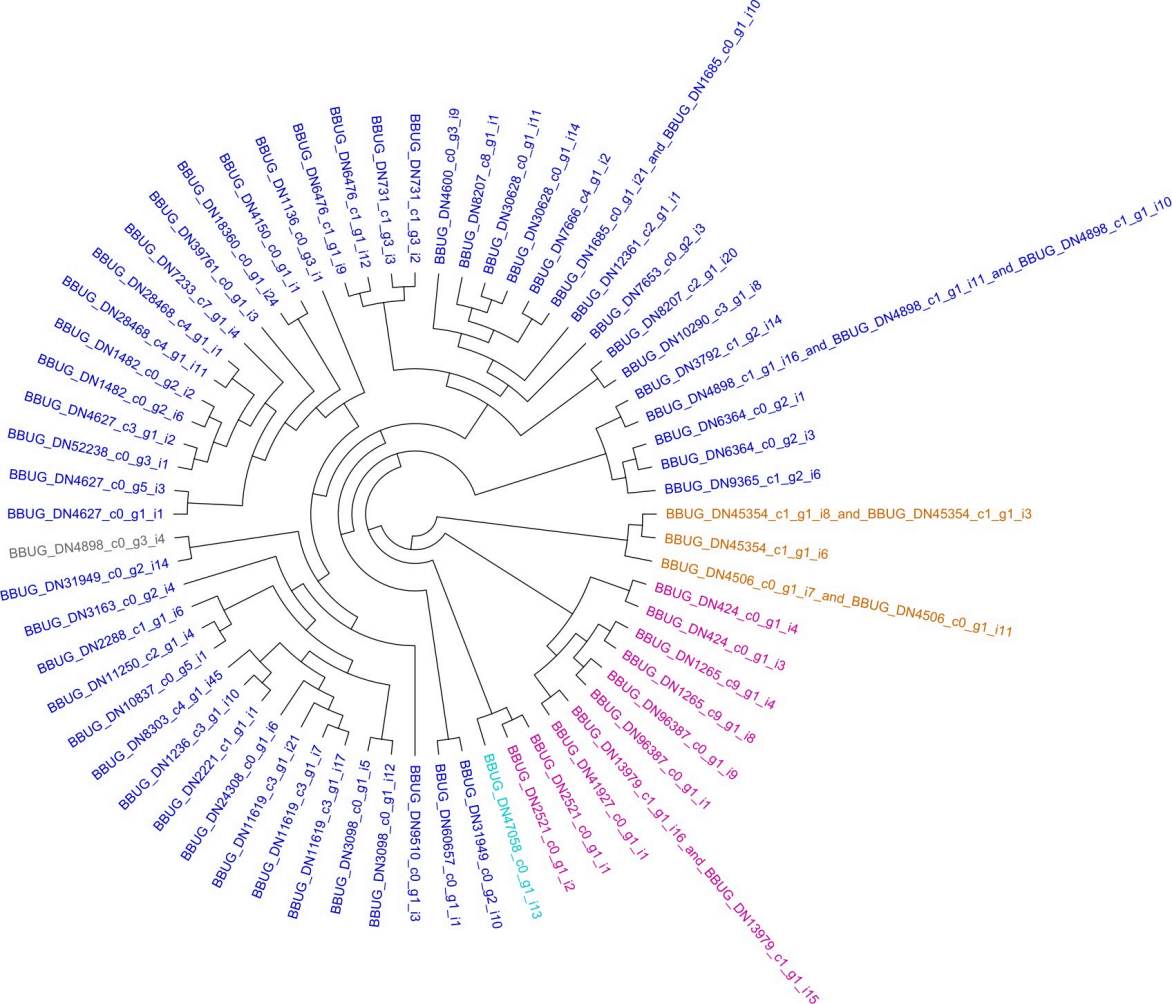
Carboxylesterase (COE) enzymes from *B*. *hilaris*. Cladogram of bagrada bug COEs distilled from the comprehensive, ten-taxa phylogram presented in S3 Fig. COE classes are connoted using leaf coloring as follows: royal blue ~ β-esterases, purple ~ neuroligins, brown ~ acetylcholinesterases, turquoise ~ neurotactins, and gray ~ palmitoleoyl COE NOTUM.

Prior annotation information was used to compile a set of 196 reference hemipteran carboxylesterase (COE) protein sequences from four species (*H*. *halys* = 79 sequences, *M*. *histrionica* = 62, *C*. *lectularius* = 31 and *D*. *citri* = 24). The *B*. *hilaris* transcriptome was compared against these using tBLASTn [48] with default parameters, which identified 64 bagrada bug COE instances. These hemipteran COEs were combined with 320 coleopteran COE sequences identified in Sparks et al. (2020) [29] (*A*. *glabripennis* = 82, *L*. *decemlineata* = 58, *A*. *vittatum* = 75, *T*. *castaneum* = 36 and *D*. *virgifera virgifera* = 69). The total set of 580 insect COEs (available in Supplementary Data as “SuppFile2.COE.fpa.txt”) was aligned and used to calculate a maximum likelihood tree as described above for GSTs, using the coleopteran branching patterns determined in S2 Fig of Sparks et al. (2020) [29] as a guide tree. The phylogeny was visualized as described above (see S3 Fig). Coleopteran nodes were purged from the comprehensive phylogeny to produce a Hemiptera-only COE phylogram (see S4 Fig) and *B*. *hilaris* nodes were similarly distilled from the phylogeny, the branching pattern of which is presented in the cladogram of Fig 2.

### Cytochrome P450 gene family analysis

**Fig 3:**
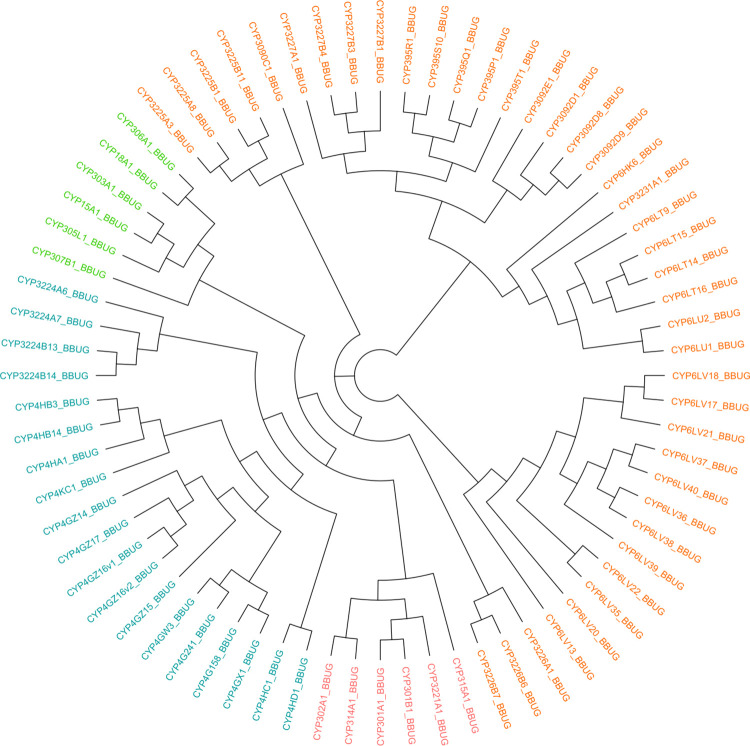
Cytochrome P450 monooxygenase (CYP) enzymes from *B*. *hilaris*. Cladogram of bagrada bug CYPs distilled from the comprehensive, ten-taxa phylogram presented in S5 Fig. CYP clans are indicated using leaf coloring: lime green ~ CYP2, orange ~ CYP3, turquoise ~ CYP4, and pink ~ Mito.

A set of 391 reference cytochrome P450 monooxygenase (CYP) protein sequences, identified from prior annotation information, was obtained for four hemipterans (*H*. *halys* = 126 sequences, *M*. *histrionica* = 85, *C*. *lectularius* = 55 and *D*. *citri* = 125). The *B*. *hilaris* transcriptome was compared against these using tBLASTn [48] with default parameters, which identified 72 bagrada bug CYP instances. These bagrada bug genes were named according to BLAST comparisons with previously named insect CYPs. All of the above hemipteran CYPs were combined with 513 coleopteran CYP sequences previously analyzed by the authors in Sparks et al. (2020) [29] (*A*. *glabripennis* = 102, *L*. *decemlineata* = 81, *A*. *vittatum* = 95, *T*. *castaneum* = 128 and *D*. *virgifera virgifera* = 107). The total set of 977 insect CYPs (which included one instance from the citrus long-horned beetle, *Anoplophora chinensis*; see Supplementary Data file “SuppFile3.CYP.fpa.txt”) was multiply aligned and used to construct a maximum likelihood phylogeny using the coleopteran branching patterns determined in S3 Fig of Sparks et al. (2020) [29] as a guide (see S5 Fig). Coleopteran nodes were purged from the comprehensive phylogeny as described above, thereby producing a Hemiptera-only CYP phylogram (see S6 Fig). Similarly, *B*. *hilaris* nodes were distilled from the phylogeny, the branching pattern of which is presented in the cladogram of Fig 3.

### Sex pheromone biosynthetic pathway analysis

Exemplary juvenile hormone and terpene-based sex pheromone biosynthetic pathway components from *M*. *histrionica*, *H*. *halys* and other taxa, presented in Tables 5 and 6 of Sparks et al. (2017) [30], were used to probe the bagrada bug transcriptome using tBLASTx or tBLASTn, as appropriate. A particular *B*. *hilaris* transcript exhibiting the greatest similarity to canonical farnesyl diphosphate synthases (USDA-ARS_BBUG.413657, designated as “FDPS-like 4”) unexpectedly contained a stop codon (TAA) at nucleotide position 421. However, this appears to have been due to an assembly error, and the correct codon should have been GAA, encoding a glutamic acid residue: all sequenced reads were mapped to this transcript in a sample-specific manner using bwa (v0.7.17-r1188, [53]); biological replicates within a sample type were combined, sorted and marked for duplicates using the Picard Toolkit (v2.25.7, https://broadinstitute.github.io/picard/); and sample-specific variants were called on read pileups using bcftools (v1.16, [54]). Mapped reads were also visualized using IGV (v2.10.2, [55]). All evidence indicated the nucleotide in question should have been G rather than T (results not shown), and so the transcript’s coding sequence was manually edited and used for downstream analysis.

A listing of closest homologs to known reference enzyme sequences is provided in Table 2. Farnesyl diphosphate synthase (FDS)-like sequences identified from bagrada bug and functionally characterized homologs from *M*. *histrionica*, *H*. *halys*, and *Nezara viridula* were combined and phylogenetically analyzed with methods similar to those used for the GST, COE and CYP gene family analyses.

**Table 2:** Transcripts encoding terpene biosynthesis-related enzymes. Cases for which the closest homolog of a *B*. *hilaris* transcript observed in *H*. *halys* was not among the query sequences used to identify that *B*. *hilaris* enzyme are indicated with bold and italicized font (note that *M*. *histrionica* mRNA sequences were not used as queries).

Enzyme Name	Query Sequences (multiple taxa)	Transcript Identified (*Bagrada hilaris*)	Closest Homolog (*Murgantia histrionica*)	Closest Homolog (*Halyomorpha halys*)
Acetoacetyl-CoA thiolase	XM_014419845, XM_014386017, XM_015512081	USDA-ARS_BBUG.113114	GECQ01446782.1	XM_014419845.1
HMG-CoA synthase	XM_014416338	USDA-ARS_BBUG.325845	GECQ01419521.1	XM_014416338.1
HMG-CoA reductase	X70034, XM_014424783, XM_014391838	USDA-ARS_BBUG.287418	GECQ01421664.1	XM_014424783.1
Mevalonate kinase	XM_014416757, XM_014391202, GEDC01029638	USDA-ARS_BBUG.136619	GECQ01227208.1	XM_014416757.1
Phosphomevalonate kinase	XM_014416475, XM_014398812, GECZ01001991, GEBQ01010256	USDA-ARS_BBUG.197385	GECQ01270718.1	XM_014416475.1
Diphosphomevalonate decarboxylase	XM_018479978, XM_014434730, XM_014399537, GEBQ01002905	USDA-ARS_BBUG.91689	GECQ01428021.1	***XM_014434716*.*1***
IDP Isomerase	XM_014415973, GECU01023093, AK417896	USDA-ARS_BBUG.406793	GECQ01092242.1	XM_014415973.1
FDP Synthase-like 1	XP_014289225	USDA-ARS_BBUG.554346	GECQ01420512.1	***XM_014420697*.*1*** ^(^^^^^)^
FDP Synthase-like 2	XP_014289225	USDA-ARS_BBUG.554360	GECQ01420512.1	***XM_014420697*.*1***
FDP Synthase-like 3	XP_014289225	USDA-ARS_BBUG.554349	GECQ01420512.1	***XM_014420697*.*1***
FDP Synthase-like 4	XP_014289225	USDA-ARS_BBUG.413657	GECQ01414919.1	***XM_014420915*.*1***
Farnesyl diphosphatase	NP_572760	*No homologs evident*	*No homologs evident*	*No homologs evident*
Farnesol dehydrogenase	XP_014292348	USDA-ARS_BBUG.590697	GECQ01376501.1	***XM_014431039*.*1*** ^(^^&^^)^
Farnesal dehydrogenase	KC243495	USDA-ARS_BBUG.620599	GECQ01079642.1	***XM_014437214*.*1***
Juvenile hormone acid methyltransferase	XP_014293044	USDA-ARS_BBUG.85661	GECQ01519496.1	XM_014437558.1
Methyl farnesoate epoxidase	XP_014283057	USDA-ARS_BBUG.763913	GECQ01517163.1	XM_014427571.1

^(^)^ XM_014420697.1 corresponds to *H*. *halys* protein XP_014276183.1 (*H*. *halys* TPS1, QBK50746.1); protein XP_014289225.1 (*H*. *halys* TPS2, QBA82488.1) is encoded by XM_014433739.1

^(&)^ XM_014431039.1 corresponds to *H*. *halys* protein XP_014286525.1; protein XP_014292348.1 is encoded by XM_014436862.1

### RNAi and viral transcript analysis

The set of top transcript hits against NCBI NR was inspected for proteins ordinarily associated with RNAi-related pathways, including the terms “dicer,” “r2d2,” “argonaute,” “sid,” “aubergine,” “tarbp,” “loquacious,” “piwi,” “helicase” and “rdrp.” To identify transcripts of potential viral origin, the assembled transcriptome was compared with a database of 13,345 viral reference sequences compiled by the International Committee on Taxonomy of Viruses (ICTV; version VMR-200721-MSL36) [56, 57] using BLASTn. A complementary approach was pursued in which raw reads were independently assembled and post-processed for viral content characterization using version 3.15.5 of the rnaviralSPAdes pipeline [58, 59]. Results from these independent analyses were then compared by manual inspection.

### Growth- and development-related transcripts

Transcripts with top hits matching key genes related to growth and development were identified by searching for the following terms among annotations: “ecdysone”, “hedgehog”, “insulin-like growth factor”, “juvenile-hormone”, “target of rapamycin”, and “wnt”. The list of transcripts was then filtered by only retaining those that were transcribed from genes observed to be differentially expressed.

## Results

### Assembly, quantitative and qualitative analyses identify differential gene expression

The global RNA-Seq assembly resulted in 725,320,098 bases assembled into 973,957 putative unique transcripts emitted from 742,910 distinct genes. A total of 268,705 distinct transcripts exhibited one or more significant hits to the NCBI NR database (only the top-scoring hit for each such query was retained). Gene-level expression estimation indicated that 741,036 genes exhibited non-zero total read counts. Among those, 67,697 exhibited differential expression in at least one comparison between differing sample condition types; 25,860 such differentially expressed genes (DEGs) emitted one or more transcripts exhibiting a match to NCBI NR (see supplemental file “SuppFile4.BBUG_DE_genes-and-tcts.xlsx”). The three most abundantly up- and down-regulated transcripts observed by DESeq2 in each pairwise comparison of life stage (or sex) are presented in Tables 3 and 4, respectively. More detailed listings, including RSEM-inferred, gene-level TPM expression values, are tabulated on the sheets “most_up-reg” and “most_down-reg” of the supplemental file, “SuppFile4.BBUG_DE_genes-and-tcts.xlsx”. Transcript-level TPM expression values, as well as Pfam and GO term annotations, are presented for DEG-associated transcripts on the “DEG-assoc_transcripts” sheet of this supplemental file, also.

**Table 3:** The three most abundantly up-regulated genes observed in all pairwise combinations of life stages/ sexes.

Comparison	Gene ID	Log_2_(Fold change)	NCBI NR hit
2^nd^ vs 4^th^	BBUG_DN4658_c13_g2	19.9032	*None*
	BBUG_DN65212_c0_g2	14.9613	*None*
	BBUG_DN5872_c1_g1	10.6484	XP_014279718.1 cuticle protein 19-like [*H*. *halys*]
2^nd^ vs Female	BBUG_DN46337_c0_g3	25.6603	XP_014294506.1 uncharacterized protein LOC106692826 [*H*. *halys*]
	BBUG_DN925_c15_g2	25.4535	*None*
	BBUG_DN145492_c0_g1	21.2933	*None*
2^nd^ vs Male	BBUG_DN46337_c0_g1	26.6957	XP_014294506.1 uncharacterized protein LOC106692826 [*H*. *halys*]
	BBUG_DN46337_c0_g5	25.1154	XP_014294506.1 uncharacterized protein LOC106692826 [*H*. *halys*]
	BBUG_DN15130_c9_g1	23.7786	*None*
2^nd^ vs Eggs	BBUG_DN46302_c6_g1	23.0275	*None*
	BBUG_DN2890_c42_g1	19.8496	XP_024215573.1 uncharacterized protein LOC112210438 isoform X1 [*H*. *halys*]
	BBUG_DN7513_c2_g1	19.0793	XP_014273630.1 uncharacterized protein LOC106679154 isoform X2 [*H*. *halys*]
4^th^ vs Female	BBUG_DN46337_c0_g3	24.2365	XP_014294506.1 uncharacterized protein LOC106692826 [*H*. *halys*]
	BBUG_DN925_c15_g2	24.1319	*None*
	BBUG_DN145492_c0_g1	20.8477	*None*
4^th^ vs Male	BBUG_DN23421_c3_g2	28.9187	*None*
	BBUG_DN46337_c0_g1	23.6505	XP_014294506.1 uncharacterized protein LOC106692826 [*H*. *halys*]
	BBUG_DN15130_c9_g1	22.1051	*None*
4^th^ vs Eggs	BBUG_DN28895_c0_g1	35.2034	*None*
	BBUG_DN65012_c0_g1	25.7611	*None*
	BBUG_DN46302_c6_g1	23.9533	*None*
Female vs Male	BBUG_DN23421_c3_g2	32.9720	*None*
	BBUG_DN46337_c0_g1	19.1140	XP_014294506.1 uncharacterized protein LOC106692826 [*H*. *halys*]
	BBUG_DN46337_c0_g5	17.4937	XP_014294506.1 uncharacterized protein LOC106692826 [*H*. *halys*]
Eggs vs Female	BBUG_DN925_c15_g2	24.2717	*None*
	BBUG_DN46337_c0_g3	23.2512	XP_014294506.1 uncharacterized protein LOC106692826 [*H*. *halys*]
	BBUG_DN925_c27_g2	21.2050	*None*
Eggs vs Male	BBUG_DN23421_c3_g2	33.9406	*None*
	BBUG_DN46337_c0_g1	24.3665	XP_014294506.1 uncharacterized protein LOC106692826 [*H*. *halys*]
	BBUG_DN14163_c12_g1	23.1351	XP_014241385.2 uncharacterized protein LOC106662089 [*Cimex lectularius*]

**Table 4:** The three most abundantly down-regulated genes observed in all pairwise combinations of life stages/ sexes.

Comparison	Gene ID	Log_2_(Fold change)	NCBI NR hit
2^nd^ vs 4^th^	BBUG_DN39844_c0_g7	-21.6146	*None*
	BBUG_DN2275_c0_g1	-12.4775	XP_024219591.1 uncharacterized protein LOC106689347, partial [*H*. *halys*]
	BBUG_DN27614_c2_g1	-12.0261	XP_024214673.1 uncharacterized protein LOC112210215 [*H*. *halys*]
2^nd^ vs Female	BBUG_DN39844_c0_g7	-22.3856	*None*
	BBUG_DN65012_c0_g1	-21.6667	*None*
	BBUG_DN23421_c3_g2	-20.6975	*None*
2^nd^ vs Male	BBUG_DN39844_c0_g7	-21.8852	*None*
	BBUG_DN65012_c0_g1	-21.8001	*None*
	BBUG_DN28895_c0_g1	-19.9114	*None*
2^nd^ vs Eggs	BBUG_DN39844_c0_g7	-23.5686	*None*
	BBUG_DN23421_c3_g2	-21.6661	*None*
	BBUG_DN4864_c0_g1	-16.9750	XP_014286374.1 uncharacterized protein LOC106687161 [*H*. *halys*]
4^th^ vs Female	BBUG_DN65212_c0_g2	-23.3434	*None*
	BBUG_DN4658_c13_g2	-18.9350	*None*
	BBUG_DN9731_c0_g2	-13.2662	XP_014290817.1 cathepsin B [*H*. *halys*]
4^th^ vs Male	BBUG_DN4658_c13_g2	-19.2406	*None*
	BBUG_DN65212_c0_g2	-15.1927	*None*
	BBUG_DN104850_c0_g1	-14.5384	XP_014284189.1 CLIP domain-containing serine protease 2 isoform X1 [*H*. *halys*]
4^th^ vs Eggs	BBUG_DN4658_c13_g2	-22.3432	*None*
	BBUG_DN65212_c0_g2	-17.1122	*None*
	BBUG_DN4879_c0_g1	-16.9262	*None*
Female vs Male	BBUG_DN925_c15_g2	-18.4571	*None*
	BBUG_DN46337_c0_g3	-16.2915	XP_014294506.1 uncharacterized protein LOC106692826 [*H*. *halys*]
	BBUG_DN145492_c0_g1	-15.7622	*None*
Eggs vs Female	BBUG_DN65012_c0_g1	-34.1789	*None*
	BBUG_DN28895_c0_g1	-33.2772	*None*
	BBUG_DN46302_c6_g1	-20.9980	*None*
Eggs vs Male	BBUG_DN28895_c0_g1	-38.3256	*None*
	BBUG_DN65012_c0_g1	-34.3123	*None*
	BBUG_DN46302_c6_g1	-21.6672	*None*

### Glutathione S-transferase gene family is typical of the Hemiptera

The bagrada bug comprises an array of GSTs typical of hemipterans (see Fig 1, S1 and S2 Figs): Theta (n = 4), Delta (n = 3), Sigma (n = 16), microsomal (n = 3), prostaglandin E synthase (n = 1) and C-terminal domain-containing GSTs (n = 1) were observed. The bagrada bug transcriptome did not appear to possess copies of Omega- or Epsilon-class sequences. Indeed, no Omega-class GSTs are apparent among any of the hemipteran species analyzed, nor were any hemipteran proteins placed among clades for the Epsilon-type GSTs identified among the Coleoptera (see S1 and S2 Figs).

### Carboxylesterase gene family analysis indicates a diverse set of β-esterases

The transcriptome contained instances of sequences placed across several different classes of COEs including β-esterases (n = 49), neuroligins (n = 10), acetylcholinesterases (n = 3), neurotactins (n = 1) and palmitoleoyl COE NOTUM (n = 1). Interestingly, a palmitoleoyl COE NOTUM gene was not specified by the bed bug genome annotation [60] and no ortholog among harlequin bug transcripts was evident, either. This subfamily consists of single-copy genes in all other taxa except bagrada bug, where an identical protein sequence appears to be encoded by two separate transcript isoforms.

### Cytochrome P450 gene family exhibits gene bloom in CYP6LV subfamily

The distribution of *B*. *hilaris* cytochrome P450 enzymes across the four canonical CYP clans was consonant with that observed in other insects (see Table 5): CYP2 (six sequences; ~8.3% of all CYPs), CYP3 (41 sequences; ~56.9%), CYP4 (19 sequences; ~26.4%), and mito (six sequences; ~8.3%). Similar to observations in the harlequin and brown marmorated stink bugs [30, 31], the CYP3 clan dominates in the bagrada bug, constituting a preponderance of its P450s and exhibiting a large gene bloom within its CYP6LV subfamily (12 sequences in *B*. *hilaris*, as compared with eight sequences in *M*. *histrionica* and 20 in *H*. *halys*).

**Table 5:** Cytochrome P450 clan-specific gene counts in select hemipteran and coleopteran species. For each species, absolute gene counts per clan are indicated and percentages relative to the respective species’ total CYP count are shown in parentheses.

Species	CYP2	CYP3	CYP4	Mito	Total
*Bagrada hilaris*	6 (8.33)	41 (56.94)	19 (26.39)	6 (8.33)	72
*Murgantia histrionica*	7 (8.24)	43 (50.59)	29 (34.12)	6 (7.06)	85
*Halyomorpha halys*	6 (4.76)	74 (58.73)	40 (31.75)	6 (4.76)	126
*Cimex lectularius*	6 (10.91)	32 (58.18)	10 (18.18)	7 (12.73)	55
*Diaphorina citri*	28 (22.40)	25 (20.00)	57 (45.60)	15 (12.00)	125
*Acalymma vittatum*	7 (7.37)	58 (61.05)	24 (25.26)	6 (6.32)	95
*Diabrotica virgifera virgifera*	7 (6.54)	63 (58.88)	29 (27.10)	8 (7.48)	107
*Leptinotarsa decemlineata*	8 (9.88)	40 (49.38)	21 (25.93)	12 (14.81)	81
*Anoplophora glabripennis*	7 (6.86)	54 (52.94)	29 (28.43)	12 (11.76)	102
*Tribolium castaneum*	8 (6.25)	69 (53.91)	42 (32.81)	9 (7.03)	128

### Enzymatic components of sex pheromone biosynthetic pathway identified

**Fig 4:**
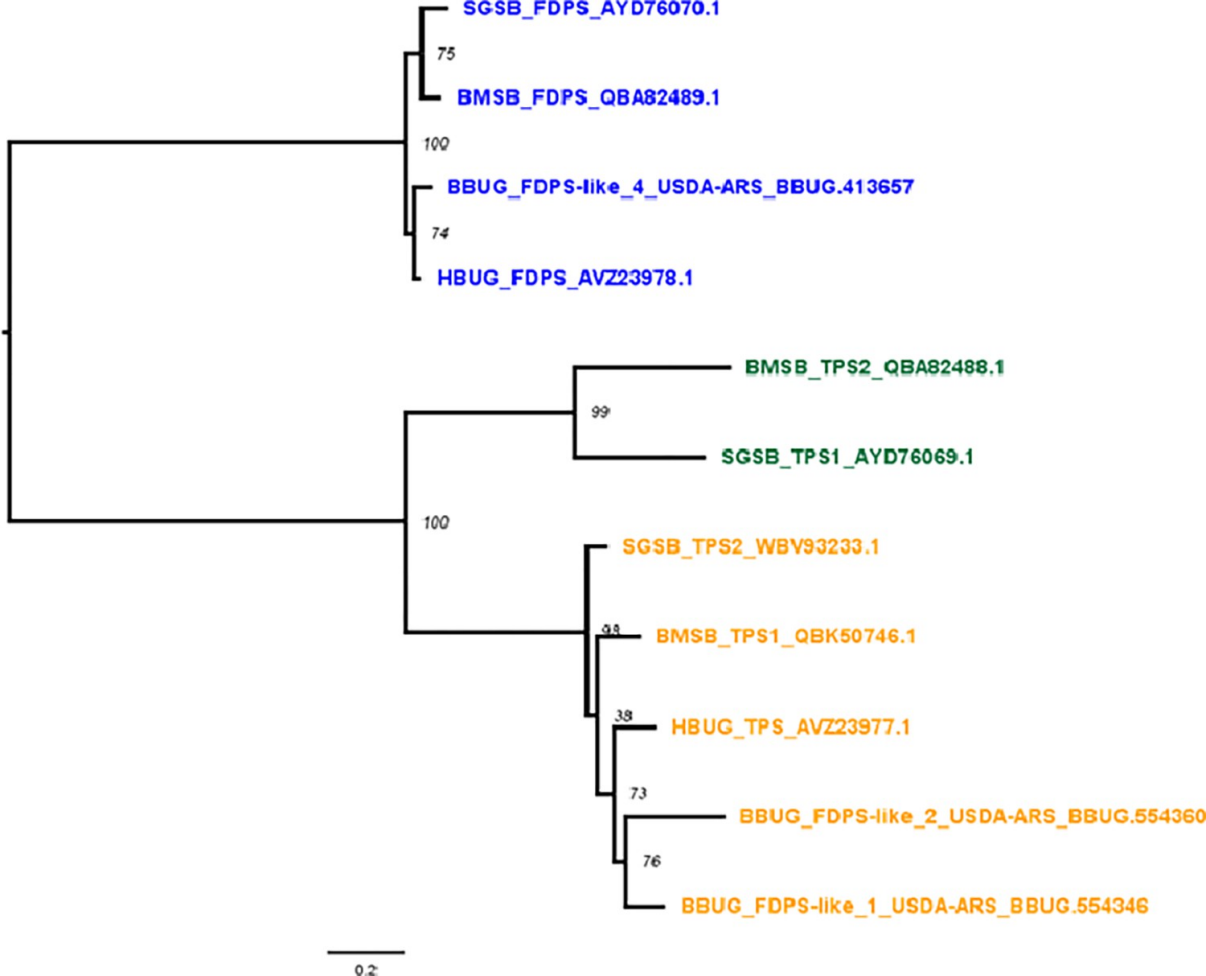
Hemipteran farnesyl diphosphate synthase (FDPS) and FDPS-like TPS (terpene synthase) phylogeny. Leaf node identifiers are prefixed by species of origin: BBUG ~ *Bagrada hilaris* (bagrada bug), BMSB ~ *Halyomorpha halys* (brown marmorated stink bug), HBUG ~ *Murgantia histrionica* (harlequin bug), and SGSB ~ *Nezara viridula* (southern green stink bug). FDPS and TPS subfamilies are shown with leaf color coding: blue ~ canonical FDPS, yellow ~ TPS-a type clade (sesquipiperitol synthase-like), and green ~ TPS-b type clade. Leaf accessions correspond to NCBI protein database accession numbers for characterized hemipteran proteins or *B*. *hilaris* transcripts encoding the protein sequences. The phylogram was rooted to the canonical FDPS clade. Branch lengths are based on a JTT model of protein evolution and the scale bar denotes estimated amino acid substitutions per site. Node support from 3,000 bootstrap replicates is displayed in italic font.

The bagrada bug transcriptome contains transcripts from all genes required for juvenile hormone biosynthesis, including a homolog of canonical FDPSs identified in other stink bugs: USDA-ARS_Bagrada_hilaris.413657 (BBUG_FDPS-like 4, bit score of 640 relative to *H*. *halys* exemplar sequence XP_014276401.1) (Table 2, Fig 4). The amino acid sequence of BBUG_FDPS-like 4 is 95% identical to that of the FDPS enzyme from *M*. *histrionica* (S1 Table). Three additional FDPS-like transcripts were detected: USDA-ARS_Bagrada_hilaris.554346 (BBUG_FDPS-like 1, bit score = 583), USDA-ARS USDA-ARS_Bagrada_hilaris.554360 (BBUG_FDPS-like 2, bit score = 517), and USDA-ARS_Bagrada_hilaris.554349 (BBUG_FDPS-like 3, bit score = 387) (Table 2, Fig 4).

### RNAi pathway gene presence and at least four viral sequences observed

Concerning RNAi-related genes, transcripts encoding the RISC-associated components Dicer (29 transcripts were annotated as such), Loquacious (four transcripts) and Argonaute (44 transcripts), as well as homologs of Tarbp2 (eight transcripts), various RNA helicases (1,570 transcripts) and PIWI or PIWI-like proteins (27 transcripts) were seen. No transcripts with evident homology to Aubergine, R2D2, Sid-1 or Sid-2 were detected. Twelve transcripts thought to encode RNA-dependent RNA polymerase activity were observed, though these appear to correspond to Pol components of retroviral elements.

A total of 15,348 *B*. *hilaris* transcripts exhibited hits with ICTV-compiled reference viral sequences. The ten most frequently encountered viral reference gene hits are displayed in Table 6. A total of 1,336 *B*. *hilaris* transcripts exhibited a top hit to genome segment Circle3 of *Cotesia congregata* PDV (GenBank identifier AJ632306.1). The viralComplete post-processing module of the rnaviralSPAdes pipeline suggested 131 transcripts existed in the RNA-Seq data corresponding to full-length viral genomes of 35 distinct genomes (see the “vc_scaffolds_result” sheet of the supplemental file, “SuppFile5.BBUG_viral_analysis.xlsx”). Scrutinous manual inspection, however, suggested that only four of these represented legitimate viral sequences: ‘NODE_64529_length_1779_cov_207.165885’ (1,779 bp, matching NC_032134.1—Beihai barnacle virus 13 strain BHTH16173 segment Seg 2 hypothetical protein gene, complete cds), ‘NODE_4708_length_6419_cov_264.954460’ and ‘NODE_9018_length_5069_cov_284.669736’ (6,419 bp and 5,069 bp, respectively, both matching NC_030296.1—Diaphorina citri densovirus, complete genome) and ‘NODE_1872_length_8408_cov_25.924055’ (8,408 bp, matching NC_033852.1—Wuhan insect virus 22 strain arthropodmix13806 segment Seg 2 hypothetical protein gene, complete cds). These four assembled scaffolds are provided in supplementary information as “SuppFile6.virus.fna.txt”.

**Table 6:** Ten most frequently encountered ICTV-compiled viral reference genes corresponding to top hits for distinct *B*. *hilaris* transcripts. The “Hit count” column indicates the number of unique bagrada bug transcripts whose top hit corresponded to the “Viral reference” gene shown. The “Ref description” column provides details about the viral gene.

Hit count	Viral reference	Ref description
1,336	AJ632306.1	Cotesia congregata virus complete genome, segment Circle3
1,280	AE006468.2	*Salmonella enterica* subsp. *enterica* serovar Typhimurium str. LT2, complete genome
829	KY442063.1	Staphylococcus phage Andhra, complete genome
362	GU244497.1	Cafeteria roenbergensis virus BV-PW1, complete genome
275	AF250284.1	Amsacta moorei entomopoxvirus, complete genoma
257	HQ336222.2	Acanthamoeba polyphaga mimivirus, complete genoma
197	HF679134.1	Mythimna separata entomopoxvirus ’L’, complete genoma
195	JN572067.1	Iaco virus strain BeAn314206 nucleocapsid protein gene, complete cds
182	AF063866.1	Melanoplus sanguinipes entomopoxvirus ’O’ isolate Tucson, complete sequence
176	KX758335.1	*Bubalus bubalis* isolate DZN1 mitochondrion, complete genome

### Growth and development genes

A total of 301 transcripts associated with genes related to insect growth and development were recovered. After filtering for transcripts associated with differentially expressed genes, a total of 166 transcripts was retained. Of these, 10 were associated with ecdysone, 16 with hedgehog, 38 with insulin-like growth factors, 51 with juvenile hormone, 17 with target of rapamycin, and 34 with wnt (see supplementary file, “SuppFile8.BBUG_growth_DE.xlsx”).

## Discussion

The gene families phylogenetically analyzed here were selected given their likely roles in xenobiotic detoxification. Several sequences have been identified that may be involved in insecticide resistance, including Delta-class GSTs [34], acetylcholinesterases [61] and the CYP6 family of the CYP3 clan [36]. The transcriptome resources contributed here can be used to help design future studies to more specifically understand whether and how these various genes are involved in insecticide resistance, as well as to identify potential mechanisms for use in circumventing resistance that may develop.

Glutathione S-transferase enzymes are implicated in insect adaptation to chemical stressors in the environment, including insecticides [62, 63]. The suite of GSTs encoded by the bagrada bug appears largely unremarkable for the Hemiptera in general and pentatomids in particular. The relatively large number of Sigma GSTs identified here (n = 16) is consistent with results seen in other species in the family Pentatomidae [31, 32]. Given that this large number of Sigma GSTs is not necessarily observed in other hemipterans, it is possible that this pattern is specific to Pentatomidae [32], although transcriptomic and/or genomic sampling of additional taxa within the group will be needed to test this hypothesis.

No Omega-class GSTs were observed among any hemipteran species considered here, nor were any GST proteins originating from the Hemiptera placed among clades for coleopteran Epsilon-class GSTs. These results are consistent with observations that Epsilon-class GSTs appear unique to the Holometabola [64]. How widespread Omega-class GSTs are among the Hemiptera is not yet well resolved, however. For instance, these enzymes are apparently absent in the pea and green peach aphids (*Acyrthosiphon pisum* and *Myzus persicae*, respectively; [65]), although present in the bird cherry-oat aphid, *Rhopalosiphum padi* [66]. (Note, however, that this latter publication asserts an Omega GST is in fact present in *A*. *pisum* [66].) In any case, all evidence contemplated in this work suggests Omega GSTs are absent from the infraorder Pentatomomorpha.

Homologs for coleopteran C-terminal domain-containing GSTs of unknown function were identified in the Hemiptera. Although these appear to be single-copy among beetles, they are apparently multi-copy among true bugs. The datasets analyzed for the bagrada and harlequin bugs (as well as striped cucumber beetle) are limited to transcriptomes—and thus not especially reliable for inferring copy number—yet these findings nevertheless suggest multiple copies exist among the Hemiptera. Moreover, the bed bug and brown marmorated stink bug instances, as per genomic data, suggest at least two copies apiece (albeit encoding identical translation products, perhaps suggesting recent, possibly independent gene duplications).

β-esterases can have a role in a variety of metabolic pathways including those involved in organophosphate resistance, as well as sex pheromone and juvenile hormone functions [33]. Across hemipterans in this study as well as others, there is considerable variation in the number of β-esterases, with some species having as few as one and others having more than 20 [49]. The large number of β-esterases observed here (n = 49) is indicative of a possible gene bloom, although more study will be needed to verify this and to understand how it may affect the evolutionary trajectory of the species.

Two separate transcript isoforms encoding identical protein sequences for a palmitoleoyl COE NOTUM gene [67] were observed in bagrada bug, in contrast to its being single-copy in all other taxa considered here except the harlequin and bed bugs, for which no copies have yet been identified. As mentioned above, transcriptome evidence often makes determination of copy number infeasible, and so it is possible that this gene is indeed multi-copy in bagrada bug. Other possibilities include alternative splicing in UTRs of pre-mRNAs transcribed from a single locus or—perhaps arguably more likely—that one of these bagrada bug transcripts is misassembled. Whole-genome sequencing and/or experimental investigations (e.g., Southern blotting) will be needed to resolve this matter.

The distribution of *B*. *hilaris* cytochrome P450 monooxygenases across the four canonical CYP clans was generally unremarkable relative to observations made in other insects. Furthermore, a substantial fraction (over half) of the bagrada bug’s P450s are contained in the CYP3 clan, and a large gene bloom within its CYP6LV subfamily was inferred; similar observations have been made in the harlequin and brown marmorated stink bugs by the authors [30, 31]. The CYP3 clan has been noted to have a particularly dynamic evolutionary history across insects with high rates of gene gain [32]. Much of this gain occurs in localized areas of the genome referred to as gene clusters, although an annotated reference genome would be required to verify this in the focal species.

Although not used for the GST phylogeny, previously published gene family phylogenies for coleopteran COEs and CYPs were used as guide trees for the respective phylogenies constructed here. Hemipteran sequences were not included in these guide trees and hence were freely able to be placed anywhere on a newly constructed tree [50, 68]. Using a guide tree can be an effective method when constructing large phylogenies and use of a guide tree is unlikely to drastically affect tree topology [69, 70]. To demonstrate that guide tree usage did not have a large effect on tree topology, additional phylogenies were generated without using guide trees and the treedist program of PHYLIP [71] was used to calculate Robinson-Foulds distances (which assess differences in tree topologies without accounting for branch lengths [72]).

The use of a guide tree in both the COE and CYP datasets did not alter overall topology in either tree in a major way, and both approaches recovered similar results (see S7 and S8 Figs, respectively). The key distinction between the two approaches to tree construction was the presence of several seemingly misplaced sequences in the unguided trees. These misplaced sequences were typically found on unusually long branches and nestled amongst sequences of different sub-families. For instance, DCIT_XP_026682768.1_neuroligin-2-like is found on a long branch in the unguided tree (see S7 Fig) with no other nearby neuroligins. However, when a guide tree is used this sequence is instead found on a shorter branch, properly placed amongst several other neuroligins (see S3 Fig). BLAST analysis also verified that sequences sister to DCIT_XP_026682768.1_neuroligin-2-like are more similar on the tree constructed using the guide tree than the tree constructed without it (results not shown). Additionally, Robinson-Foulds distances calculated between the guided and unguided trees also suggest both approaches produced similar topologies (see S2 Table in the supplement for additional discussions).

Transcript expression levels for each *B*. *hilaris* gene identified in this study in the GST, COE and CYP gene families are organized into tables and colorized by expression intensity in the supplemental file, “SuppFile7.BBUG_detox_heat_maps.xlsx”. Some of these genes clearly exhibited preferential expression in specific life stages and/or sexes, although further experimental studies would be necessary to determine their specific physiological roles in those biological contexts.

Among the FDPS-like transcripts identified, the BBUG_FDPS-like 3 transcript appears to be a fragment of the BBUG_FDPS-like 1 sequence and may be a mRNA pseudomolecule derived from the same gene. Interestingly, the amino acid sequences of BBUG_FDPS-like 1 and 2 were found to be more closely related to FDPS-type terpene synthases (TPSs) that make sesquipiperitol, the sesquiterpene precursor of the *M*. *histrionica* and *H*. *halys* pheromones (Fig 4 and S1 Table) [73, 74]. These TPSs belong to the pentatomid TPS-a type clade (Fig 4), which represents one of two distinct TPS subfamilies in pentatomids [74]. In particular, the amino acid sequence encoded by the BBUG_FDS-like 1 transcript is 80% identical to that of the *M*. *histrionica* TPS (AVZ23977.1; S1 Table) suggesting that a functional sesquipiperitol synthase and a terpene pheromone pathway may also exist in bagrada bug. However, biochemical efforts to identify a terpene-based pheromone similar to that found in *H*. *halys* and *M*. *histrionica*, have not yet been successful (Jocelyn Millar, University of California, Riverside, CA, USA, pers. comm., 25 Feb. 2023). It should be noted that transcripts of a functionally active sesquipiperitol synthase have also been identified in *N*. *viridula* (*N*. *viridula* TPS2) despite the presence of a sesquipiperitol-independent terpene pheromone biosynthetic route in this species [74]. It is therefore possible that sesquipiperitol synthase-like genes serve other functions in pentatomids independent of their role in pheromone biosynthesis. Molecular genetic and biochemistry experiments will be needed to determine the exact function of the identified *B*. *hilaris* gene products.

The potential for RNAi-mediated knockdown of target genes in insects as a means for molecular biopesticide-based control has become increasingly attractive in recent years [75, 76]. Various protein machinery necessary for an RNAi response was observed in the bagrada bug, as has also been found in closely related stink bugs (e.g., *M*. *histrionica* [30] and *H*. *halys* [31]), demonstrating good prospects for the use of RNAi-based biopesticides in this species, as well as demonstrating the importance of using only highly species-specific dsRNA gene targets to minimize risks of off-target effects.

Many of the viral reference sequences in Table 6 are large double strand DNA viruses (including polydnaviruses (PDV), entomopoxviruses and mimiviruses), and matches in these instances may correspond to sequences of viral homologs of host cellular genes or repetitive sequences, for instance. However, all but two of the 1,336 *B*. *hilaris* transcripts exhibiting a top hit to genome segment Circle3 of *Cotesia congregata* PDV mapped to within positions 3720–4330 of the reference sequence, an unannotated region encoding neither repetitive elements nor protein coding genes; BLASTn comparisons against NCBI NT indicated this content was similar to sequences present in the genomes of coleopteran (e.g., *Malthinus flaveolus*) and lepidopteran (e.g., *Melitaea athalia*) insects. Recently, Heisserer et al. (2023) [77] showed that many PDV genes have been acquired by lepidopterans, sometimes accidentally, through host integration motif-mediated horizontal transfer. Discerning the evolutionary history of PDV or other DNA virus sequences expressed in *B*. *hilaris* or other hemipterans, and whether similar horizontal transfer may have been involved, is an area for further research. Many bagrada bug transcripts suggested to be of viral origin by rnaviralSPAdes appeared to be false positives, often comprising ORFs that matched to insect-specific sequences. Four of its predicted viral transcripts, however, did appear to represent legitimate viral sequences and these will be the subject of future experimental characterization.

Differential gene expression analysis revealed 166 DEG-associated transcripts implicated in insect growth and development. The majority of these were annotated as juvenile hormone, which is unsurprising given the great importance of this molecule in governing both metamorphosis and reproduction [78]. Although some of these transcripts exhibited quite abundant expression specific to only one or a few sample categories, in many cases they exhibited minimal expression levels (see “SuppFile8.BBUG_growth_DE.xlsx”). This may be due to only very transient expression of such genes and/or that they are transcribed at very limited levels in the cell. Additional experimental work is needed to gain a better understanding of the importance of these transcripts and more generally of the pathways governing growth and development.

The transcriptomic resources provided by this study should help to better understand key biological aspects of the bagrada bug, which is among the most important and emerging agricultural insect pests. The phylogenetic analyses presented herein provide a rich resource to facilitate functional genetic studies of important enzymes germane to insecticide detoxification and pheromone synthesis in *B*. *hilaris* and other agriculturally important insects. These data will also be useful towards annotating the bagrada bug genome, whose sequencing is currently in progress by the USDA-ARS.

## Supporting information

S1 Table Percent amino acid residue identities shared between B. hilaris, H. halys, M. histrionica, and N. viridula FDPS and FDPS-like TPS protein sequences based on a MUSCLE alignment. (XLSX)

S2 Table (XLSX)

S1 Fig (PDF)

S2 Fig (PDF)

S3 Fig (PDF)

S4 Fig (PDF)

S5 Fig (PDF)

S6 Fig (PDF)

S7 Fig (PDF)

S8 Fig (PDF)
