# Supplementary material for: Transcriptomic resources for Bagrada hilaris (Burmeister), a widespread invasive pest of Brassicales
Source: PLoS One. 2024 Dec 27;19(12):e0310186. doi: 10.1371/journal.pone.0310186 (PMC11676520; doi:10.1371/journal.pone.0310186)

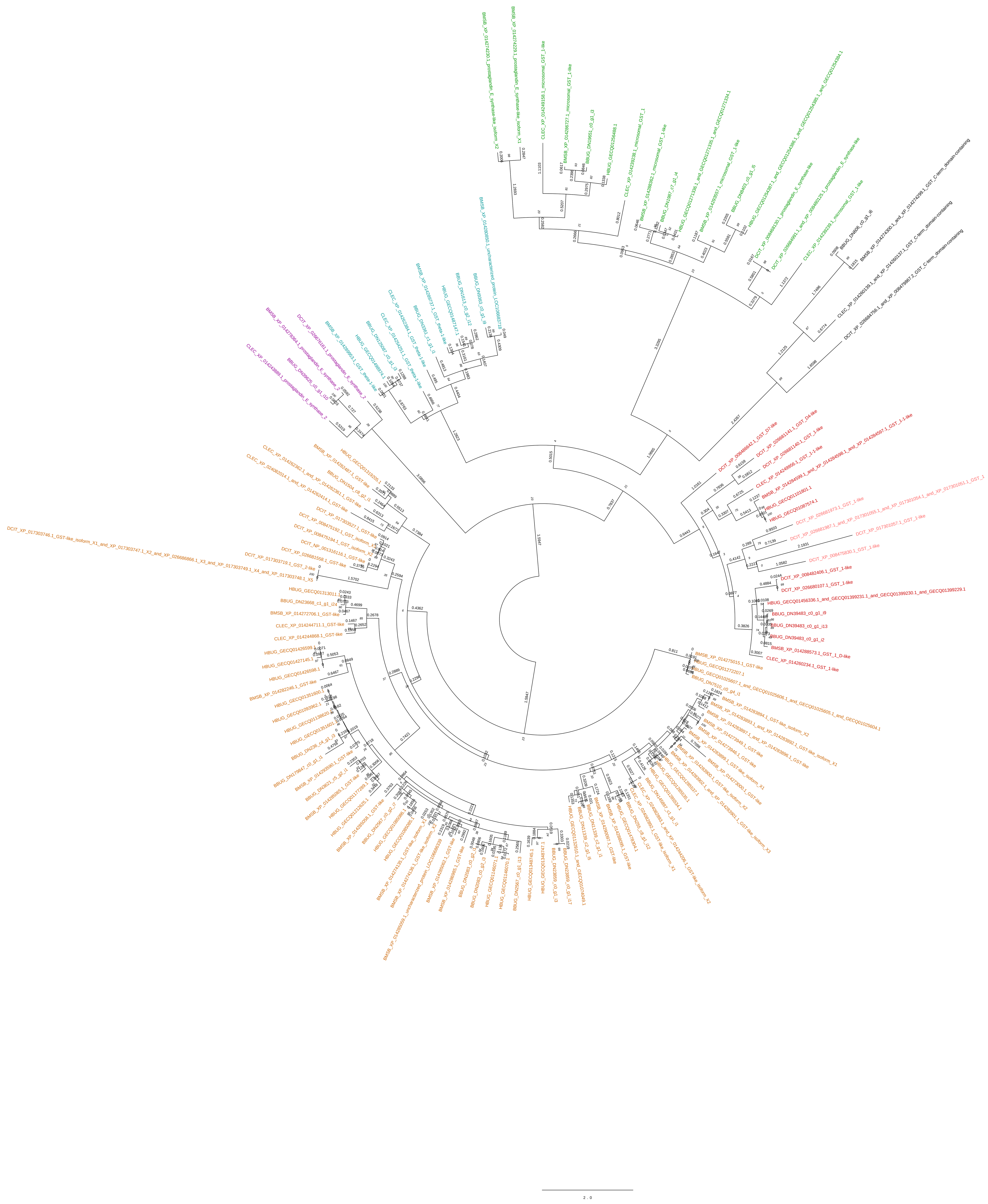

Supplement: S2 Fig — (PDF) [file pone.0310186.s004.pdf]
